# Investigate the Metabolic Reprogramming of *Saccharomyces cerevisiae* for Enhanced Resistance to Mixed Fermentation Inhibitors via ^13^C Metabolic Flux Analysis

**DOI:** 10.1371/journal.pone.0161448

**Published:** 2016-08-17

**Authors:** Weihua Guo, Yingying Chen, Na Wei, Xueyang Feng

**Affiliations:** 1 Department of Biological Systems Engineering, Virginia Polytechnic Institute and State University, Blacksburg, VA 24061, United States of America; 2 Department of Civil and Environmental Engineering and Earth Sciences, University of Notre Dame, Notre Dame, IN 46556, United States of America; National Renewable Energy Laboratory, UNITED STATES

## Abstract

The fermentation inhibitors from the pretreatment of lignocellulosic materials, e.g., acetic acid and furfural, are notorious due to their negative effects on the cell growth and chemical production. However, the metabolic reprogramming of the cells under these stress conditions, especially metabolic response for resistance to mixed inhibitors, has not been systematically investigated and remains mysterious. Therefore, in this study, ^13^C metabolic flux analysis (^13^C-MFA), a powerful tool to elucidate the intracellular carbon flux distributions, has been applied to two *Saccharomyces cerevisiae* strains with different tolerances to the inhibitors under acetic acid, furfural, and mixed (i.e., acetic acid and furfural) stress conditions to unravel the key metabolic responses. By analyzing the intracellular carbon fluxes as well as the energy and cofactor utilization under different conditions, we uncovered varied metabolic responses to different inhibitors. Under acetate stress, ATP and NADH production was slightly impaired, while NADPH tended towards overproduction. Under furfural stress, ATP and cofactors (including both NADH and NADPH) tended to be overproduced. However, under dual-stress condition, production of ATP and cofactors was severely impaired due to synergistic stress caused by the simultaneous addition of two fermentation inhibitors. Such phenomenon indicated the pivotal role of the energy and cofactor utilization in resisting the mixed inhibitors of acetic acid and furfural. Based on the discoveries, valuable insights are provided to improve the tolerance of *S*. *cerevisiae* strain and further enhance lignocellulosic fermentation.

## Introduction

The production of biofuels and other bio-based chemicals from renewable resources has been widely applied to overcome the limitation of non-renewable fossil fuel energy and the challenge of global warming. Due to the fast growth and high biofuel productivity, *Saccharomyces cerevisiae* is one of the most important workhorses in producing biofuels from various carbohydrates of biomass with high abundance and low cost. However, several fermentation inhibitors generated from the pretreatment of lignocellulosic materials, such as acetic acid [1], furfural [2], and furan [3], seriously impair the biofuel production by repressing or even stopping the cell growth of *S*. *cerevisiae* [4–7]. More importantly, most of these inhibitors ubiquitously co-exist in the practical fermentation process. Therefore, it is important to elucidate the general metabolic responses of *S*. *cerevisiae* to different inhibitors, especially to mixed inhibitors, to further improve stress resistance and reduce stress impairment. Constructing a general mechanism of inhibition is important, as in reality mixed inhibitors are hard to avoid. By identifying a common target, it is thus more likely to find a way to solve the problem in practice.

A number of biological characterizations have been accomplished to unravel the effect of fermentation inhibitors and resistance mechanism in yeast [1, 3–5, 8, 9], and several key metabolic responses have been found to be related to the stress resistance [3, 6, 7, 10–12]. It was found that acetic acid, a fermentation inhibitor commonly presenting in the lignocellulosic hydrolysate, could lead to the fermentation arrest and suppression of ethanol production for *S*. *cerevisiae* strains when using glucose as the major substrate. The mechanisms for acetic acid inhibition have been investigated in *S*. *cerevisiae* strains [13–19]. Several strategies, such as genome screening [20–22], metabolic engineering [23–25], and evolutionary engineering [26], have successfully been developed to improve yeast resistance to acetic acid. Similarly, the mechanism of furfural inhibition has been studied for more than three decades [2, 3, 27–30]. It was found that the resistance of *S*. *cerevisiae* strains to furfural could be improved by either reducing or oxidizing the furfural to less toxic compounds [2, 27, 31]. In addition, the overexpression of the genes in pentose phosphate pathway as well as several key transcription factors has been implemented to successfully improve the tolerance to furfural in *S*. *cerevisiae* strains [25, 29].

Despite growing understanding of the biomolecular mechanisms of yeast resistance to single inhibitors (e.g., acetic acid or furfural), the general molecular basis of yeast resistance to mixed fermentation inhibitors remains unclear. Considering the fact that various inhibitors often co-exist in the hydrolysate and could cooperate with each other to become even more toxic to yeast than existing alone (i.e., synergistic stress), the knowledge on how yeast cells reprogram their metabolism in response to mixed fermentation inhibitors is of particular interests to biofuel and biochemical production. The key challenge in studying yeast resistance to mixed inhibitors lies in that the resistance phenotype usually involves very complex multi-genic regulations. Additionally, various fermentation inhibitors in the cellulosic hydrolysates usually have distinct toxicity mechanisms. As such, the reprogramming of yeast metabolism to resist mixed fermentation inhibitors is largely unknown. Recently, several pioneer studies have been accomplished on the transcriptional responses to mixed inhibitors of acetate and furfural [32]. However, metabolic reprogramming in responses to such mixed fermentation inhibitors remains unclear. In this scenario, metabolic flux analysis could provide a “common language”, i.e., intracellular metabolic flux distributions, to uncover and evaluate different inhibitory metabolic reprogramming for corresponding stress conditions. Therefore, in this study, we applied ^13^C metabolic flux analysis (^13^C-MFA), a powerful and accurate tool to demystify the intracellular metabolism [33–35], on two *S*. *cerevisiae* strains, i.e., a parent strain S-C1, and an engineered strain YC1 with improved fermentation inhibitor resistance. We used ^13^C-MFA to systematically investigate the metabolic reprogramming of the *S*. *cerevisiae* strains in four conditions, namely blank condition (without any inhibitor), acetic acid, furfural, and dual-stress conditions with both acetic acid and furfural. By analyzing the carbon flux distribution as well as the energy and cofactor utilization, we elucidated the key metabolic responses under different stress conditions. Particularly, the lack of energy and cofactor supply was found to be the main reason for the synergistic stress caused by the co-presence of acetic acid and furfural. To our best knowledge, it is the first time that ^13^C-MFA was used to study the metabolic responses of *S*. *cerevisiae* under the stress of mixed fermentation inhibitors. The discovery from this work provides valuable biological insights to further improve the yeast resistance to fermentation inhibitors, especially mixed fermentation inhibitors.

## Materials and Methods

### Plasmids, strains and media

All the strains and plasmids used in this study were summarized in Table 1. The *S*. *cerevisiae* strain SR8-trp was kindly provided by Dr. Yong-Su Jin’s lab. The parent strain (named as S-C1) and the engineered strain (named as YC1, a mutant with improved resistance to acetic acid and furfural) were obtained through the inverse metabolic engineering approach in our recent work [36]. Specifically, it has been demonstrated that overexpression of WHI2, a gene target encoding a cytoplasmatic globular scaffold protein in *S*. *cerevisiae*, improved the resistance of yeast to acetic acid dramatically. *Escherichia coli* TOP10 strain was used for gene cloning and manipulation. *E*. *coli* strains were grown in Luria-Bertani medium at 37°C and 100 μg/mL of ampicillin was added to the medium when required. Yeast strains were routinely cultivated at 30°C in YP medium (10 g/L of yeast extract and 20 g/L of peptone) or synthetic complete (SC) medium (6.7 g/L of yeast nitrogen base, 0.6 g/L complete supplement mixture) containing 20 g/L of D-glucose. SC media containing 20 g/L agar and glucose, 20 mg/L histidine and uracil and 100 mg/L leucine without tryptophan amendment was used to select transformants using TRP1 as an auxotrophic marker.

**Table 1:** Plasmids and Strains.

Plasmids and strains	Description	References
**Plasmids**		
pRS424	*TRP1*, a multicopy plasmid	[37]
pRS424-*WHI2*	pRS424 with insert of S288c yeast genomic DNA fragment chrXV: 409,259–412,369 (containing complete sequence of the *WHI2* gene)	[19]
pRS424GPD	pRS424 with GPD^a^ promoter	[37]
**Strains**		
D452-2	*MATa*, *leu2*, *his3*, *ura3*, *can1*	[38]
SR8	D452-2 expressing *XYL1*, *XYL2*, and *XKS1* through integration, evolutionary engineering in xylose-containing media, and *ALD6* deletion.	[39]
SR8-*trp*	SR8 with *TRP1* disrupted	Developed in Dr. Yong-Su Jin Lab
S-C1	SR8-*trp* harboring pRS424GPD, as a control	[19]
YC1	SR8-*trp* harboring pRS424-*WHI2*	[19]

^a^ GPD stands for Glyceraldehyde-3-phosphate dehydrogenase, encoded by the *TDH3* gene. The TDH3 promoter is often referred to as the *GPD* promoter, which is used in the pRS4XX series of expression vectors [37].

### Isotopic labeling experiment

The isotopic labeling experiments were accomplished by following a previously developed protocol [40]. In general, both the S-C1 and YC1 strains were cultured in a minimal medium with 20 g/L ^13^C-glucose (a mixture of 80% [1-^13^C] and 20% [U-^13^C] glucose) as the sole carbon source at the oxygen-limited condition. Four conditions, i.e., blank condition without any inhibitor, acetic acid stress condition with 2 g/L acetic acid, furfural stress condition with 1g/L furfural, and the dual-stress condition with 2 g/L acetic acid and 1g/L furfural, were used to culture the yeast strains to study the metabolic responses. The cell growth was monitored by OD_600_. The extracellular metabolite quantification using high performance liquid chromatograph (HPLC) was also conducted to assess the consumption of glucose and the production of ethanol, glycerol, and acetic acid (Table 2). For each of the culture conditions, the ^13^C-labeled biomass was harvested during the exponential growth phase. The cell variability was checked at OD_600_ under stress conditions. The OD_600_ was found to be linearly correlated (R^2^ = 0.99) with colony-forming units (CFU) under the stress conditions (S2 Fig), indicating the appropriateness of using OD_600_ to evaluate the cell variability.

**Table 2:** Growth of S-C1 strain and YC1 strain under different stress conditions.

Stresses	Strains	Growth rates (1/h)	Specific rates (mmol/g DCW/h)			
			Glucose	Ethanol	Acetate	Glycerol
Blank	S-C1	0.117±0.017	16.00±1.64	23.03±1.22	0.00±0.00	1.69±0.10
	YC1	0.121±0.002	14.12±0.75	19.61±0.15	0.00±0.00	1.94±0.01
Acetic acid	S-C1	0.021±0.000	6.06±0.16	8.47±0.26	-0.16±0.01	0.24±0.01
	YC1	0.039±0.000	11.10±0.00	13.31±0.25	-1.13±0.04	0.58±0.00
Furfural	S-C1	0.053±0.002	18.29±1.35	27.66±1.81	0.00±0.00	2.00±0.16
	YC1	0.053±0.002	18.02±0.25	23.98±2.52	0.00±0.00	1.94±0.06
Mixed	S-C1	0.021±0.000	2.49±0.45	2.29±0.21	-0.25±0.10	0.00±0.00
	YC1	0.025±0.000	2.68±0.34	3.42±0.22	-0.19±0.02	0.74±0.03

Following harvest, isotopomer analysis of proteinogenic amino acids was conducted as previously described [40]. In brief, the biomass was hydrolyzed using 6 M HCl (20 h at 100°C) and the amino acids were derivatized using 50 μl tetrahydrofuran and 50 μL N-(tert-butyldimethylsilyl)-N-methyl-trifluoroacetamide (Sigma-Aldrich) to form tert-butyl dimethylsilyl (TBDMS) derivatives (1h at 70°C). Gas Chromatography-Mass Spectrometry (GC-MS) was performed to analyze derivatized amino acids using a Shimadzu GC2010 GC with a SH-Rxi-5Sil column and a Shimadzu QP2010 MS. Three types of charged fragments were detected by GC-MS for various amino acids: the [M-57] ^+^ group (containing unfragmented amino acids); and the [M-159] ^+^ or [M-85] ^+^ group (containing amino acids that had lost an α-carboxyl group). For each type of fragments, the labeling patterns, i.e., mass distribution vectors (MDVs), were represented by M0, M1, M2, etc., which were fractions of non-labeled, singly-labeled, and doubly-labeled amino acids. The effects of natural isotopes on isotopomer labeling patterns were corrected by using a previously reported algorithm [41].

### Metabolic flux analysis

The metabolic flux analysis was accomplished by following a previously developed protocol [40]. In general, the MDVs were used to calculate the summed fractional labeling (SFL) values which were directly used to calculate metabolic fluxes in Biomet Toolbox 2.0 (based on MATLAB, MathWorks, Inc. MA) [42]. The central carbon metabolic model for the MFA was developed previously [40] based on the KEGG database (http://www.genome.jp/kegg/), including glycolysis pathway, pentose phosphate pathway, anaplerotic pathways, the tricarboxylic acid (TCA) cycle, and the transport reactions between different cell compartments (S1 Table). For metabolic flux analysis, flux estimation was repeated at least 50 times starting with different initial values generated by a genetic algorithm for all the fluxes to find a likely global solution. A fit of the simulated and measured SFLs was determined to be a global solution only after the solution fit was obtained at least twice using this method (S1 Fig). Based on the carbon fluxes (S2 Table), the ATP, NADH, and NADPH production and consumption rates were estimated by following previous published works [40, 43]. The unpaired Student’s t-test (*α* = 0.05) was used as the statistical method to determine the significance of the net productions of ATP and cofactors under two different conditions or of two different strains.

## Results

### Metabolic responses to acetic acid stress

**Fig 1:**
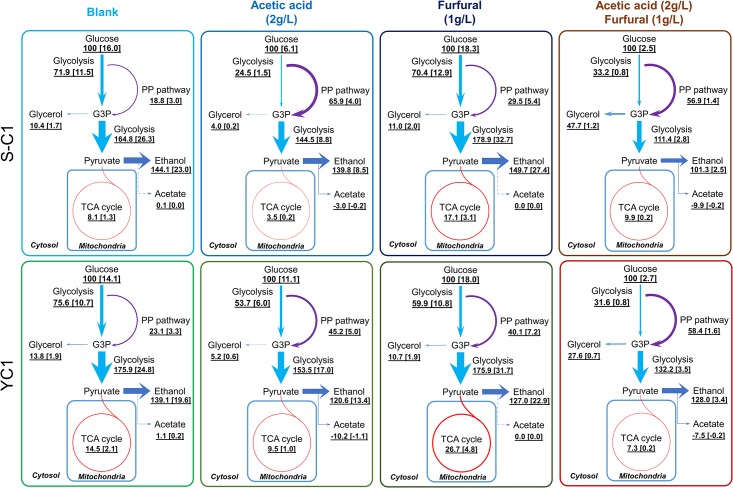
Metabolic flux distribution of the S-C1 strain and YC1 strain under different stress conditions. The values outside the bracket are relative flux values normalized to glucose uptake rates as 100. The values inside the bracket are real flux values in mmol/g/h. Abbreviations used are: G3P, glyceraldehyde 3-phosphate; TCA cycle, tricarboxylic acid cycle. The line widths are linearly correlated with the normalized flux values (glucose uptake rate as 100). The dashed line indicates the flux is zero.

**Fig 2:**
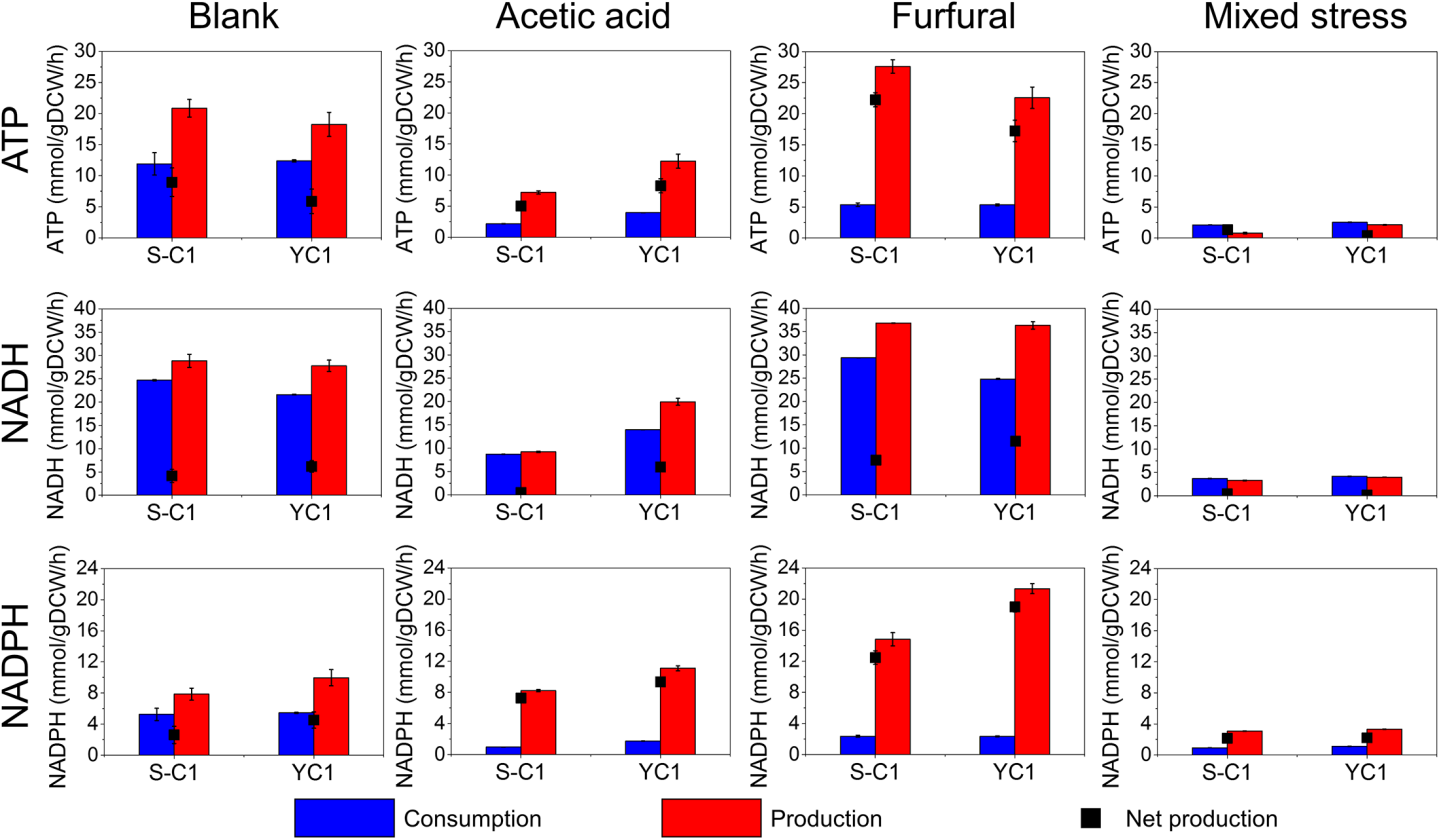
Production and consumption of cofactor and energy. The consumption (blue bar), production (red bar), and net production (black dots) of ATP, NADH, and NADPH are shown for different stress conditions. The error bars present the standard deviations, which can be too small to be seen.

When cultivating the parent *S*. *cerevisiae* strain, S-C1, under the acetic acid stress condition, the growth rate and glucose uptake rate were repressed by 80% and 60%, respectively (Table 2). Such suppression of the growth and glucose uptake was also observed in previous studies [3, 22, 44]. By comparing the flux distributions of S-C1 strain cultivated in blank and acetic acid stress conditions (Fig 1), a significant increase (33%) of carbon fluxes in the pentose phosphate pathway (PP pathway) and a dramatic decrease (66%~87%) of carbon fluxes in the glycolysis pathway were observed (Fig 1). As a result, the ethanol production decreased by >60% in the acetic acid stress condition compared to that in the blank condition. In addition, the carbon fluxes in the TCA cycle dramatically decreased (>85%) under the acetic acid stress condition, which is consistent with previous studies [13, 15]. The net production of ATP and NADH was decreased under the acetic acid stress condition due to the decreased glycolysis and TCA cycle (Fig 2). However, the NADPH net production was increased due to the increased PP pathways.

Similarly as that in the S-C1 strain, when cultivating the mutant *S*. *cerevisiae* strain, YC1, in the acetic acid stress condition, the growth rate and glucose uptake rate were depressed by 68% and 21%, respectively (Table 2). By comparing the flux distributions of YC1 strain cultivated in blank and acetic acid stress conditions (Fig 1), a significant increase (51%) of carbon fluxes in the PP pathway and a decrease (~44%) of carbon fluxes in the glycolysis pathway were observed, which decreased the ethanol production by 32%. The TCA cycle was also decreased (~50%) under the acetic acid stress condition, which was similar as that in the wild-type strain. By calculating the consumption and production of cofactors based on the flux distribution, the net production of ATP and NADH under the acetic acid stress was found not significantly different from those under the blank condition. However, the NADPH net production was increased by ~100% due to the increased PP pathways (Fig 2).

**Fig 3:**
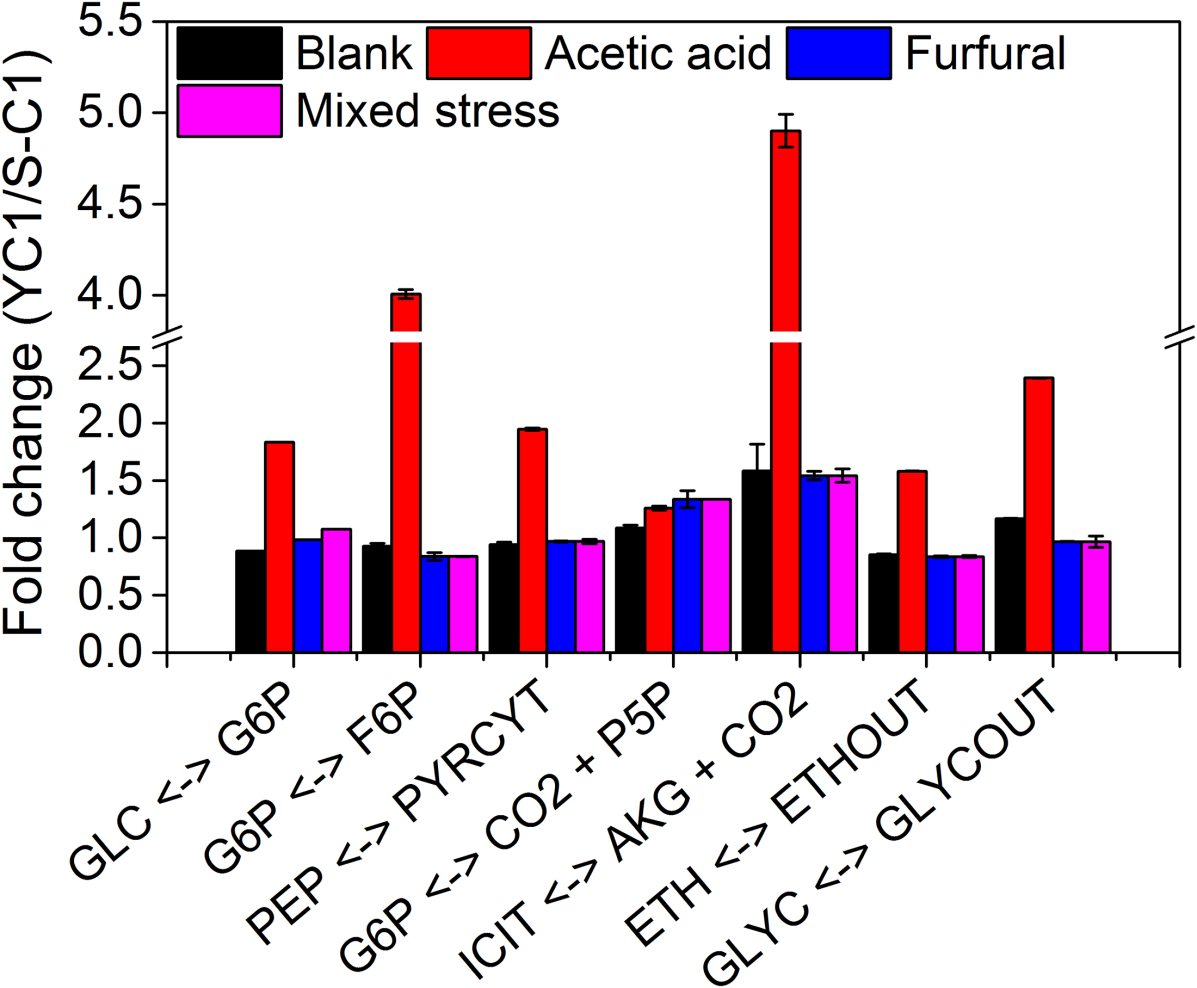
Fold changes of key fluxes between the S-C1 and YC1 strain under different stress conditions. Abbreviation: GLC, glucose; G6P, glucose-6-phosphate; F6P, fructose-6-phosphate; PEP, phosphoenolpyruvate; PYRCYT, pyruvate in cytosol; P5P, ribulose 5-phosphate; ICIT, isocitrate; AKG, α-ketoglutarate; ETH, ethanol; ETHOUT, extracellular ethanol; GLYC, glycerol; GLYCOUT, extracellular glycerol.

Comparing the metabolic responses between the S-C1 strain and YC1 strain under the acetic acid stress condition, the similar pattern of the alteration of flux distributions was observed, namely increased PP pathway, decreased glycolysis pathway, as well as the decreased TCA cycle when comparing to the blank condition (Fig 1). This indicated that both the S-C1 strain and YC1 strain responded to the acetic acid stress in a similar manner. However, the YC1 strain demonstrated better growth rate (~100% higher than the S-C1 strain, Table 2) and glucose uptake rate (~80% higher than the S-C1 strain) under the acetic acid stress condition. Such superior performance could be related to the higher activities of the glycolysis and the TCA cycle in the mutant strain (Fig 3), which produced more ATP (~50% increase in net produced ATP compared to that in the S-C1 strain, Fig 2) for relieving the oxidative stresses inflicted by acetic acid. In addition, the higher activities of PP pathway could produce more NADPH for supporting macromolecule synthesis for cell growth under acetic acid stress condition.

### Metabolic responses to furfural stress

When cultivating the *S*. *cerevisiae* S-C1 strain under the furfural stress condition, the growth rate decreased by ~50% with a slightly increased glucose uptake rate (~10%) (Table 2). Such suppressed growth but increased glucose uptake was also observed in a previous study on chemostat when feeding a high concentration of furfural [28]. By comparing the flux distributions of the S-C1 strain cultivated in blank and furfural stress condition (Fig 1), a significant increase of carbon fluxes in the PP pathway (~80%) as well as glycolysis pathway (~12%) was observed. The carbon fluxes in TCA cycle were also increased by ~135% under the furfural stress condition. The elevated activities of glycolysis, PP pathway, and the TCA cycle led to the increased net production of ATP, NADH and NADPH in the S-C1 strain under the furfural stress condition (Fig 2).

Similarly, when cultivating the *S*. *cerevisiae* YC1 strain under the furfural stress condition, the growth rate was decreased by ~56% with an increased glucose uptake rate (~30%). By comparing the flux distributions of the YC1 strain cultivated in blank and furfural stress condition (Fig 1), an increase of the carbon fluxes in the PP pathway (~118%), glycolysis pathway (~28%), and the TCA cycle (~128%) was observed under the furfural stress condition, which is similar as that in the wild-type strain. Therefore, the net production of ATP, NADH and NADPH were also increased in the strain YC1 under the furfural stress condition (Fig 2).

Comparing the metabolic responses between the resistant strain YC1 and control S-C1, the carbon flux distributions were similarly altered, i.e., the activities of the PP pathway, glycolysis pathway, TCA cycle were all elevated. Therefore, both strains responded to the furfural stress in a similar manner (Fig 3). As a result of such metabolic reprogramming, the net production of ATP and NADPH was increased. The increased ATP and NADPH production could play important roles in resisting the furfural stress in the yeast. Basically, NADPH could be used as the cofactor for alcohol dehydrogenase to convert furfural to furfuryl alcohol, a less toxic compound to *S*. *cerevisiae*, while the over-production of ATP could relieve the oxidative stress inflicted by furfural. Noticeably, the carbon fluxes in the PP pathway and the TCA cycle were higher in the strain YC1 than that in the control strain S-C1. Although this significantly improved the YC1 cell growth, the carbon loss in the PP pathway and the TCA cycle slightly decreased the ethanol yield. The observation clearly indicated a trade-off between cell growth and the ethanol production when cultivated with furfural.

### Metabolic responses to mixed fermentation inhibitors

When cultivating the *S*. *cerevisiae* S-C1 strain under the dual-stress condition with acetic acid and furfural, the growth rate and glucose uptake rate were dramatically decreased by 82% and 84%, respectively. By comparing the flux distributions of S-C1 strain under blank condition and the dual-stress condition (Fig 1), we found that all the fluxes were dramatically decreased. For example, the carbon fluxes in the PP pathway, glycolysis, and TCA cycle were decreased by ~53%, ~90%, and ~85%, respectively. Compared to the flux distributions in acetic acid stress condition or furfural stress condition, the extent of carbon flux decrease was much severe in the presence of both acetic acid and furfural. The results indicated that the mixed inhibitors of acetic acid and furfural led to synergistic stress on the S-C1 *S*. *cerevisiae* strain, i.e., the presence of both inhibitors is much more toxic than either individual inhibitor.

Similarly, when cultivating the *S*. *cerevisiae* YC1 strain under the dual-stress condition with both acetic acid and furfural, the growth rate and glucose uptake rate were dramatically decreased by 80% and 80%, respectively. The synergistic stress of acetic acid and furfural was also observed in the *S*. *cerevisiae* YC1 strain, in which the carbon fluxes in all of the metabolic pathways significantly decreased (Fig 1). The severe inhibitory effects of acetic acid and furfural mixture could be highly correlated with the energy and cofactor production. In general, for both YC1 strain and S-C1 strain, the net production of ATP, NADH and NADPH were universally suppressed (Fig 2). As a result, the oxidative stress inflicted by acetic acid and furfural could not be relieved, while on the other hand, furfural could not be sufficiently degraded to less toxic compound due to the lack of cofactor supplies (e.g., NADPH) for the alcohol dehydrogenase.

### Comparisons of yeast metabolic responses to different fermentation inhibitors

To highlight the similarities and differences of yeast metabolic responses under different inhibitor treatments, we have compared metabolic flux distributions and corresponding ATP and cofactor balances between different stress conditions. For the stress conditions with single inhibitors, i.e., acetic acid and furfural, the growth rate and glucose uptake were dramatically impaired under the acetic acid stress compared to the unstressed condition, while cell growth was impaired but glucose uptake was improved under furfural stress condition (Table 1). As a result of improved glucose uptake, ethanol production increased under furfural stress condition but decreased under acetic acid stress condition. In addition, more carbon flux was directed towards the glycolysis pathway under the furfural stress condition, while more carbon flux was redirected to the pentose phosphate pathway under acetic acid stress condition. In addition, more carbon going through the TCA cycle under furfural stress condition, but acetic acid impaired the TCA cycle dramatically. Due to the different metabolic flux distributions, the energy and cofactor productions also varied. Specifically, ATP net production under furfural stress was improved from the untreated condition. Whereas, under acetic acid stress, ATP net production was impaired slightly. NADH net productions of both inhibitors did not change dramatically. However, both inhibitors led to a significant increase in NADPH net production. The NADPH production under furfural stress condition was much higher than that of acetic acid stress condition due to the enhanced glucose uptake.

Next, we compared the metabolic responses to single inhibitors (i.e., acetic acid or furfural) with that to mixed inhibitors in order to highlight the different mechanisms used by yeast to resist single and mixed inhibitors. Compared to acetic acid stress condition, cell growth was similar to that of the mixed inhibitors, but glucose uptake was dramatically impaired under dual stress condition (>59%, Table 1). Due to the impaired glucose uptake rate, the net productions of ATP, NADH, and NADPH were also decreased dramatically under dual-stress condition compared to those under acetic acid stress only. Compared to furfural stress condition, both cell growth and glucose uptake under dual stress condition decreased by ~55% and ~85%, respectively. As a result, the impaired glucose uptake under dual-stress condition led to a decrease of net productions of ATP, NADH, and NADPH compared to those under furfural stress only. Overall, by comparing the metabolic responses between different inhibitors, it is evident that different inhibitors could lead to varied metabolic responses to resist corresponding stress conditions, as discussed below.

## Discussion

**Fig 4:**
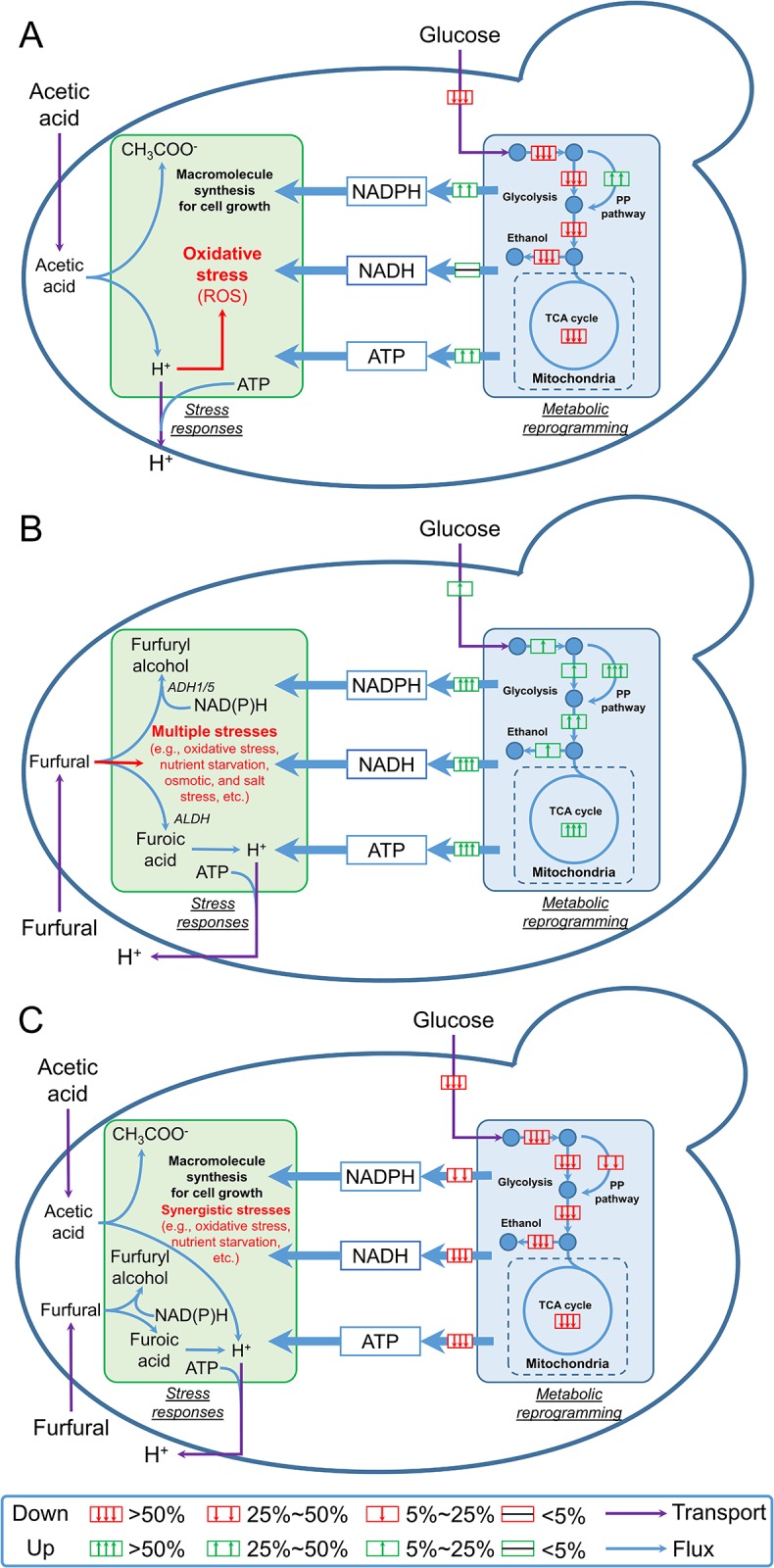
Generalized mechanisms used by *S*. *cerevisiae* strains in different stress conditions. (A): acetic acid stress condition; (B): furfural stress condition; (C): dual-stress condition; Abbreviations: ALDH, aldehyde dehydrogenase; ADH1/5, alcohol dehydrogenase 1/5, ROS, reactive oxygen species.

Based on the metabolic reprogramming under different stress conditions, we proposed the mechanisms that could be used by the *S*. *cerevisiae* strains for resisting acetic acid, furfural, or mixed inhibitors of acetic acid and furfural (Fig 4). As for the acetic acid stress, it was found that NADPH was over-produced from the PP pathway in both the S-C1 strain and the resistant YC1 strain. Such extra NADPH could be used to support the macromolecule synthesis for cell growth. Meanwhile, the ATP production could be another important factor in resisting acetic acid stress. It is well known that acetic acid could inflict oxidative stress. At the low pH (pH<4.76) when using glucose as the substrate, the acetic acid would enter the cells by facilitated diffusion in the un-dissociated form [45]. Once in the cytosol with the neutral environment, the acetic acid would release proton. The accumulation of the intracellular proton would then induce the accumulation of the reactive oxygen species (ROS) under the aerobic or oxygen-limited condition and the dysfunction of mitochondrial by hyper-activation of Ras–cAMP–PKA pathway (Fig 4A) [13]. In this scenario, the TCA cycle would be impaired severely due to the ROS accumulation and the mitochondrial dysfunction. Therefore, as shown in the metabolic flux analysis on the YC1 strain, the over-produced ATP from the second-half of the glycolysis (i.e., energy releasing steps) could relieve the oxidative stress and improve the resistance of yeast cells to the acetic acid stress. It is worth mentioning that introducing other ROS quenchers, e.g., oxygen, could be an effective approach to relieve the oxidative stresses induced by ROS, but with the risk of further reprogramming the intracellular metabolism. In this case, protons from dissociated acetic acid have been considered the major stress contributor. It is also important, however, to consider acetic acid as two isolated parts, i.e., weak acid and proton donor. Evaluating the impact of the two factors of acetic acid on yeast metabolism is beyond the scope of this work. However, we are currently designing new experiments to examine the corresponding metabolic reprogramming of yeast by comparing the metabolic fluxes of yeasts when being treated with acetic acid and another inorganic acid (e.g. HCl). In addition, it is important to point out that acetic acid could also be an endogenous byproduct of biofuel production, which could be taken as a potential inhibitor for fermentation. In this study, we have omitted the synthesis of endogenous acetic acid due to the extremely low concentration compared to the exogenous acetic acid as the inhibitor. However, metabolic responses of yeast to endogenous and exogenous acetic acids could vary. We are currently developing new experiments to address this concern.

For the furfural stress, over-production of ATP and NADPH is pivotal (Fig 4B). The stress responses inflicted by furfural are multiple, including oxidative stress, nutrient starvation, and osmotic and salt stress (Fig 4B) [27]. As consistent with previous studies [28, 29], we found a dramatic increase of the NADPH net production (>4-fold) was observed in both the S-C1 strain and the YC1 strain (Fig 2). These extra NADPH could be used by alcohol dehydrogenase to convert furfural to less toxic furfuryl alcohol. In the meantime, during aerobic or oxygen-limited conditions, furfural could be oxidized to furoic acid with the regeneration of NADH [11, 27]. Although the furoic acid is less toxic compared to furfural, the dissociation of furoic acid could increase the proton concentration inside the cell, and hence, lead to the oxidative stress. The over-produced ATP from energy-releasing steps in glycolysis and the TCA cycle could relieve the oxidative stress inflicted by furfural and improve the resistance of yeast cells. It is also worth mentioning that the central metabolism was up-regulated but cell reproduction was compromised in furfural stress condition. Such phenomenon is possibly related to the enhanced glucose uptake, a step that consumed the majority of ATP. The high demand of ATP would up-regulate energy-producing pathways such as glycolysis. However, because majority ATP was used to uptake glucose, not enough ATP could be supplied to support cell growth, which led to decreased cell growth rate in furfural stress condition.

For the dual-stress with acetic acid and furfural, the two inhibitors could introduce synergistic stress. In this scenario, the glucose transport was severely limited due to the lack of ATP caused by oxidative stress (Table 2). Consequently, the activities of all the metabolic pathways were repressed, and the energy and cofactor production was hence jeopardized. Without the sufficient supply of NADPH, enzymes such as alcohol dehydrogenase could hardly convert the inhibitors into less toxic compounds. The accumulation of these inhibitors would in return reinforce the inhibitory effects, which lead to even more severe decrease on cell growth. It was also interesting to notice that the reprogramming of metabolic fluxes in YC1 compared to that of S-C1 strain was much more dramatic in the acetic acid stress condition than that of furfural stress condition or the mixed inhibitor stress condition (Fig 3). This is consistent with how the strain YC1 was developed in our previous work[18]. Namely, the YC1 strain was initially developed and screened for improved resistance to acetic acid. However, it could be possible that when both acetic acid and furfural were present, the stress, especially the oxidative stress, could be so strong that the cell metabolism had to prioritize the resource to compensate for the ATP loss. Therefore, the engineered metabolism in the YC1 strain was not fully exploited to demonstrate superior resistance to mixed inhibitors of acetic acid and furfural.

In summary, for the stress condition with single inhibitors, *S*. *cerevisiae* strains intend to overproduce the energy and cofactors to either relieve oxidative stress or to convert inhibitors to less toxic compounds. However, in the presence of mixed inhibitors, yeast cells face difficulty in producing enough ATP and NADPH to resist the heavier stresses, which deteriorate the inhibitor resistance and lead to dramatic impairment of cell growth. Using metabolic flux analysis to study yeast stress responses provides a “common language” so that different inhibitory mechanisms could be evaluated and discussed on the same platform, which is the key to enable the correlation between different phenotypes and genotypes. The engineering strategies focusing on optimizing energy and cofactor supply would be worthy in improving yeast resistance to mixed fermentation inhibitors.

## Conclusion

In this study, we applied ^13^C-MFA to investigate the reprogramming of cell metabolism of two *S*. *cerevisiae* strains in the presence of various inhibitors. In general, *S*. *cerevisiae* strains adopt different mechanisms to resist acetic acid stress, furfural stress, and dual-stress with both acetic acid and furfural. The resistance mechanisms heavily relied on the energy and cofactor production, especially when mixed fermentation inhibitors were present. The lack of ATP and NADPH was found to be the main reason for the poor performance of *S*. *cerevisiae* strains when cultivated with both acetic acid and furfural. Based on the discovery of this study, the host engineering of *S*. *cerevisiae* strains to improve energy and cofactor synthesis could be a potential strategy to improve yeast resistance to fermentation inhibitors in hydrolysate from lignocellulosic materials.

## Supporting Information

S1 Fig Simulated and observed SFLs for S-C1 strain and YC1 strain under different stress conditions. (TIF)

S2 Fig Linear correlation between OD_600_ and CFU. (TIF)

S1 Table The central metabolic model used in this study. (DOCX)

S2 Table Metabolic fluxes of S-C1 strain and YC1 strain under different stress conditions. All the flux values were normalized to glucose uptake rates (set as 100), respectively.
(DOCX)
