# Supplementary figures and images for: Investigate the Metabolic Reprogramming of Saccharomyces cerevisiae for Enhanced Resistance to Mixed Fermentation Inhibitors via 13C Metabolic Flux Analysis
Source: PLoS One. 2016 Aug 17;11(8):e0161448. doi: 10.1371/journal.pone.0161448 (PMC4988770; doi:10.1371/journal.pone.0161448)

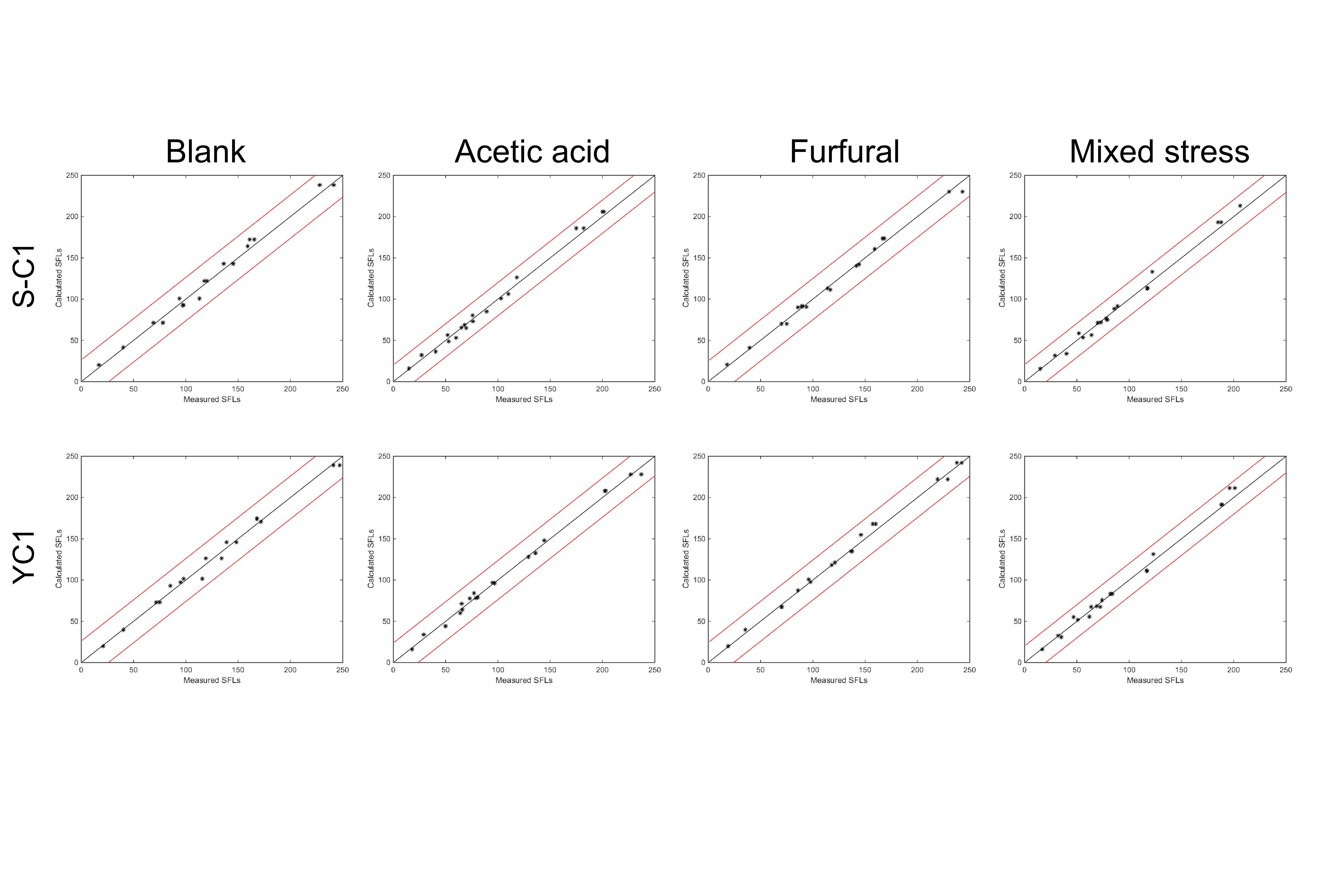

Supplement: S1 Fig — (TIF) [file pone.0161448.s001.TIF]

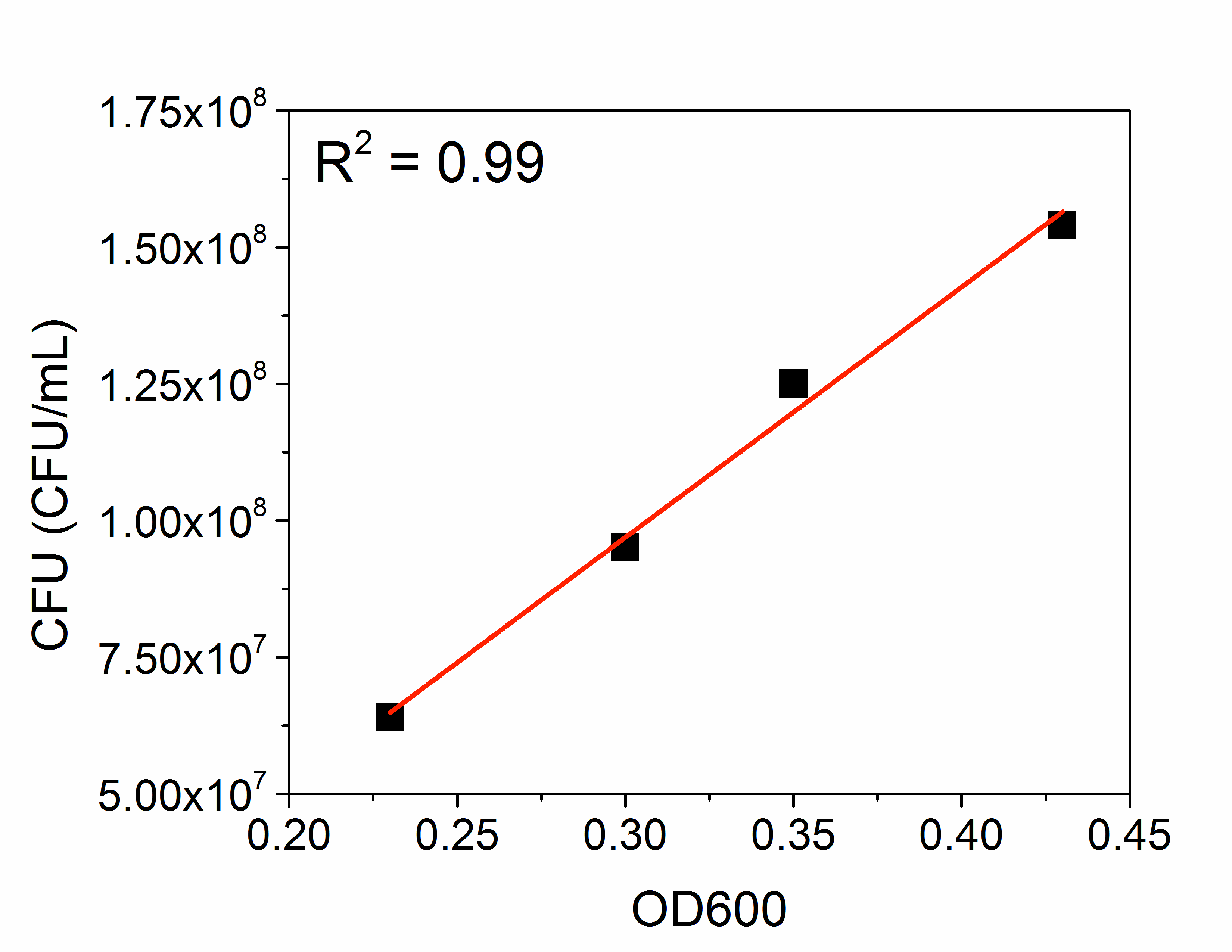

Supplement: S2 Fig — (TIF) [file pone.0161448.s002.tif]
