# Supplementary material for: Investigate the Metabolic Reprogramming of Saccharomyces cerevisiae for Enhanced Resistance to Mixed Fermentation Inhibitors via 13C Metabolic Flux Analysis
Source: PLoS One. 2016 Aug 17;11(8):e0161448. doi: 10.1371/journal.pone.0161448 (PMC4988770; doi:10.1371/journal.pone.0161448)
Supplement: S1 Table — (DOCX) [file pone.0161448.s003.docx]

**S1 Table. The central metabolic model used in this study.**

| **ID** | **Reactions** |
| --- | --- |
| v1 | GLC → G6P |
| v2 | G6P ↔ F6P |
| v3 | F6P ↔ DHAP + G3P |
| v4 | DHAP ↔ G3P |
| v5 | G3P → PEP |
| v6 | PEP ↔ PYRCYT |
| v7 | G6P → CO2 + P5P |
| v8 | P5P + P5P ↔ S7P + G3P |
| v9 | S7P + G3P ↔ F6P + E4P |
| v10 | P5P + E4P ↔ F6P + G3P |
| v12 | PYRCYT ↔ ACA + CO2 |
| v13 | ACA ↔ ETH |
| v14 | ACA ↔ ACE |
| v15 | G3P ↔ GLYC |
| v16 | ACE → ACCOACYT |
| v17 | PYRCYT + CO2 ↔ OAACYT |
| v18 | PYRMIT ↔ ACCOAMIT + CO2 |
| v19 | OAAMIT + ACCOAMIT ↔ ICIT |
| v20 | ICIT ↔ AKG + CO2 |
| v21 | AKG ↔ FUM + CO2 |
| v22 | FUM + FUM ↔ OAAMIT + OAAMIT |
| v24 | OAAMIT ↔ OAACY |
| v25 | ACCOACYT ↔ ACCOAMIT |
| v26 | PYRCYT ↔ PYRMIT |
| v27 | G3P ↔ SER |
| v28 | SER ↔ GLY + C1 |
| v29 | OAACYT ↔ THR |
| v30 | THR ↔ GLY + ACA |
| v31 | PYRMIT + CO2 ↔ OAAMIT |
| v32 | G6P → G6POUT |
| v33 | P5P → P5POUT |
| v34 | E4P → E4POUT |
| v35 | G3P → G3POUT |
| v36 | PEP → PEPOUT |
| v37 | PYRMIT → PYRMITOUT |
| v38 | PYRCYT → PYRCYTOUT |
| v39 | OAACYT → OAACYTOUT |
| v40 | AKG → AKGOUT |
| v41 | ACCOACYT → ACCOACYTOUT |
| v42 | ACCOAMIT → ACCOAMITOUT |
| v43 | SER → SEROUT |
| v44 | GLY → GLYOUT |
| v45 | C1 → C1OUT |
| v46 | THR → THROUT |
| v47 | ETH → ETHOUT |
| v48 | ACE → ACEOUT |
| v49 | GLYC → GLYCOUT |
| v50 | CO2 → CO2OUT |
