# Supplementary material for: Investigate the Metabolic Reprogramming of Saccharomyces cerevisiae for Enhanced Resistance to Mixed Fermentation Inhibitors via 13C Metabolic Flux Analysis
Source: PLoS One. 2016 Aug 17;11(8):e0161448. doi: 10.1371/journal.pone.0161448 (PMC4988770; doi:10.1371/journal.pone.0161448)
Supplement: S2 Table — All the flux values were normalized to glucose uptake rates (set as 100), respectively. (DOCX) [file pone.0161448.s004.docx]

**S2 Table.** **Metabolic fluxes of S-C1 strain and YC1 strain under different stress conditions.** All the flux values were normalized to glucose uptake rates (set as 100), respectively.

| **ID** | **Blank** | | | | **Acetic acid** | | | | **Furfural** | | | | **Mixed** | | | |
| --- | --- | --- | --- | --- | --- | --- | --- | --- | --- | --- | --- | --- | --- | --- | --- | --- |
|  | **S-C1** | | **YC1** | | **S-C1** | | **YC1** | | **S-C1** | | **YC1** | | **S-C1** | | **YC1** | |
|  | **Flux** | **STD** | **Flux** | **STD** | **Flux** | **STD** | **Flux** | **STD** | **Flux** | **STD** | **Flux** | **STD** | **Flux** | **STD** | **Flux** | **STD** |
| v1 | 100.0 | 0.0 | 100.0 | 0.0 | 100.0 | 0.0 | 100.0 | 0.0 | 100.0 | 0.0 | 100.0 | 0.0 | 100.0 | 0.0 | 100.0 | 0.0 |
| v2 | 71.9 | 1.3 | 75.6 | 1.2 | 24.5 | 0.6 | 53.7 | 1.1 | 70.4 | 2.2 | 59.9 | 0.6 | 33.2 | 0.0 | 31.6 | 0.0 |
| v3 | 84.5 | 1.3 | 91.1 | 0.9 | 65.2 | 1.7 | 82.2 | 1.6 | 90.1 | 0.7 | 86.6 | 0.2 | 71.1 | 0.1 | 70.5 | 0.0 |
| v4 | 84.5 | 1.3 | 91.1 | 0.9 | 65.2 | 1.7 | 82.2 | 1.6 | 90.1 | 0.7 | 86.6 | 0.2 | 71.1 | 0.1 | 70.5 | 0.0 |
| v5 | 164.8 | 2.6 | 175.9 | 1.7 | 145.1 | 2.3 | 163.5 | 3.4 | 179.0 | 0.6 | 175.9 | 0.2 | 112.0 | 2.3 | 132.2 | 0.6 |
| v6 | 164.8 | 2.6 | 175.9 | 1.7 | 144.5 | 1.5 | 153.5 | 3.4 | 178.9 | 0.7 | 175.9 | 0.2 | 111.4 | 2.1 | 132.2 | 0.6 |
| v7 | 18.8 | 0.0 | 23.1 | 0.6 | 65.9 | 1.2 | 45.2 | 0.4 | 29.5 | 2.2 | 40.1 | 0.6 | 56.9 | 0.0 | 58.4 | 0.0 |
| v8 | 6.3 | 0.0 | 7.7 | 0.2 | 20.6 | 0.9 | 14.3 | 0.3 | 9.9 | 0.7 | 13.4 | 0.2 | 18.9 | 0.0 | 19.5 | 0.0 |
| v9 | 6.3 | 0.0 | 7.7 | 0.2 | 20.6 | 0.9 | 14.3 | 0.3 | 9.9 | 0.7 | 13.4 | 0.2 | 18.9 | 0.0 | 19.5 | 0.0 |
| v10 | 6.3 | 0.0 | 7.7 | 0.2 | 20.1 | 0.3 | 14.3 | 0.3 | 9.8 | 0.7 | 13.4 | 0.2 | 18.9 | 0.0 | 19.4 | 0.0 |
| v12 | 147.5 | 2.7 | 148.9 | 8.0 | 140.2 | 0.1 | 120.6 | 0.0 | 154.6 | 2.3 | 138.6 | 4.9 | 101.4 | 0.9 | 126.7 | 0.7 |
| v13 | 144.1 | 0.7 | 139.1 | 0.5 | 139.8 | 0.1 | 120.6 | 0.1 | 149.7 | 0.6 | 127.0 | 0.6 | 101.3 | 0.8 | 128.0 | 0.4 |
| v14 | 3.4 | 3.5 | 9.8 | 8.4 | 0.5 | 0.1 | 0.0 | 0.0 | 4.9 | 2.7 | 11.6 | 4.8 | 0.2 | 0.1 | 0.0 | 0.0 |
| v15 | 10.4 | 0.0 | 13.8 | 0.0 | 4.0 | 0.0 | 5.2 | 0.0 | 11.0 | 0.0 | 10.7 | 0.0 | 47.7 | 2.3 | 27.6 | 0.3 |
| v16 | 3.3 | 3.4 | 8.7 | 7.6 | 3.4 | 0.2 | 10.3 | 0.0 | 4.9 | 2.6 | 11.6 | 4.8 | 10.1 | 0.1 | 7.5 | 0.2 |
| v17 | 1.7 | 1.7 | 0.0 | 0.0 | 0.8 | 0.3 | 9.5 | 4.4 | 0.0 | 0.0 | 11.6 | 0.6 | 4.7 | 0.8 | 3.3 | 0.8 |
| v18 | 9.2 | 6.3 | 12.5 | 7.4 | 0.1 | 0.1 | 0.4 | 0.0 | 12.2 | 2.4 | 15.6 | 4.5 | 0.2 | 0.1 | 0.0 | 0.0 |
| v19 | 8.1 | 1.4 | 14.5 | 2.3 | 3.5 | 0.1 | 9.5 | 2.1 | 17.1 | 0.5 | 26.7 | 0.6 | 9.9 | 0.5 | 7.3 | 0.3 |
| v20 | 8.1 | 1.4 | 14.5 | 2.3 | 3.5 | 0.1 | 9.5 | 2.1 | 17.1 | 0.5 | 26.7 | 0.6 | 9.9 | 0.5 | 7.3 | 0.3 |
| v21 | 0.0 | 0.0 | 0.4 | 0.0 | 2.5 | 0.1 | 8.5 | 2.1 | 5.0 | 0.0 | 5.0 | 0.0 | 8.4 | 0.5 | 5.6 | 0.3 |
| v22 | 0.0 | 0.0 | 0.2 | 0.0 | 1.3 | 0.0 | 4.2 | 1.1 | 2.5 | 0.0 | 2.5 | 0.0 | 4.2 | 0.2 | 2.8 | 0.2 |
| v24 | -1.7 | 1.7 | 0.3 | 0.3 | -0.7 | 0.4 | 2.0 | 5.1 | 0.0 | 0.0 | -11.6 | 0.6 | 3.0 | 1.7 | 0.3 | 1.2 |
| v25 | 3.0 | 3.4 | 7.2 | 8.3 | 3.4 | 0.2 | 10.3 | 0.0 | 4.9 | 2.6 | 11.6 | 4.8 | 10.1 | 0.0 | 7.5 | 0.2 |
| v26 | 15.6 | 6.3 | 27.0 | 9.0 | 2.1 | 1.9 | 13.4 | 5.1 | 24.3 | 2.3 | 25.7 | 4.3 | 5.2 | 1.8 | 2.0 | 0.9 |
| v27 | 0.0 | 0.0 | 0.1 | 0.1 | 0.0 | 0.0 | 0.0 | 0.0 | 0.1 | 0.1 | 0.0 | 0.0 | 0.0 | 0.0 | 0.5 | 0.1 |
| v28 | 0.0 | 0.0 | 0.1 | 0.0 | 0.0 | 0.0 | 0.0 | 0.0 | 0.1 | 0.1 | 0.0 | 0.0 | 0.0 | 0.0 | 0.2 | 0.2 |
| v29 | 0.0 | 0.0 | 0.1 | 0.1 | 0.1 | 0.1 | 6.0 | 5.3 | 0.0 | 0.0 | 0.0 | 0.0 | 0.3 | 0.2 | 1.4 | 0.3 |
| v30 | 0.0 | 0.0 | 0.0 | 0.0 | 0.1 | 0.1 | 0.0 | 0.0 | 0.0 | 0.0 | 0.0 | 0.0 | 0.1 | 0.0 | 1.4 | 0.3 |
| v31 | 6.4 | 2.0 | 14.4 | 2.5 | 0.3 | 0.4 | 3.0 | 5.1 | 12.1 | 0.5 | 10.1 | 0.3 | 4.5 | 1.3 | 2.0 | 0.9 |
| v32 | 9.2 | 1.3 | 1.2 | 0.9 | 9.6 | 0.8 | 1.1 | 1.5 | 0.0 | 0.0 | 0.0 | 0.0 | 9.9 | 0.0 | 10.0 | 0.0 |
| v33 | 0.0 | 0.0 | 0.0 | 0.0 | 4.6 | 3.1 | 2.4 | 0.4 | 0.0 | 0.0 | 0.0 | 0.0 | 0.1 | 0.1 | 0.0 | 0.0 |
| v34 | 0.0 | 0.0 | 0.0 | 0.0 | 0.4 | 0.8 | 0.0 | 0.1 | 0.0 | 0.0 | 0.0 | 0.0 | 0.0 | 0.0 | 0.0 | 0.0 |
| v35 | 0.0 | 0.0 | 0.0 | 0.0 | 1.5 | 2.9 | 10.0 | 0.0 | 0.0 | 0.1 | 0.0 | 0.0 | 1.3 | 1.1 | 0.2 | 0.3 |
| v36 | 0.0 | 0.0 | 0.0 | 0.0 | 0.5 | 1.0 | 10.0 | 0.0 | 0.1 | 0.3 | 0.0 | 0.0 | 0.6 | 0.5 | 0.0 | 0.0 |
| v37 | 0.0 | 0.0 | 0.1 | 0.2 | 1.7 | 1.7 | 10.0 | 0.0 | 0.0 | 0.1 | 0.0 | 0.0 | 0.5 | 0.7 | 0.0 | 0.0 |
| v38 | 0.0 | 0.0 | 0.0 | 0.0 | 1.5 | 0.3 | 10.0 | 0.0 | 0.0 | 0.1 | 0.0 | 0.0 | 0.2 | 0.1 | 0.2 | 0.2 |
| v39 | 0.0 | 0.0 | 0.2 | 0.3 | 0.0 | 0.0 | 5.5 | 2.1 | 0.0 | 0.0 | 0.0 | 0.0 | 7.4 | 1.7 | 2.2 | 0.9 |
| v40 | 8.1 | 1.4 | 14.2 | 2.3 | 1.0 | 0.0 | 1.0 | 0.0 | 12.1 | 0.5 | 21.7 | 0.6 | 1.5 | 0.6 | 1.7 | 0.3 |
| v41 | 0.2 | 0.2 | 1.5 | 1.8 | 0.0 | 0.0 | 0.0 | 0.0 | 0.0 | 0.0 | 0.0 | 0.0 | 0.0 | 0.0 | 0.0 | 0.0 |
| v42 | 4.1 | 4.4 | 5.2 | 3.4 | 0.0 | 0.0 | 1.2 | 2.1 | 0.0 | 0.0 | 0.4 | 0.9 | 0.3 | 0.4 | 0.2 | 0.2 |
| v43 | 0.0 | 0.0 | 0.0 | 0.1 | 0.0 | 0.0 | 0.0 | 0.0 | 0.0 | 0.0 | 0.0 | 0.0 | 0.0 | 0.0 | 0.3 | 0.3 |
| v44 | 0.0 | 0.0 | 0.1 | 0.1 | 0.1 | 0.1 | 0.0 | 0.0 | 0.1 | 0.1 | 0.0 | 0.0 | 0.1 | 0.0 | 1.6 | 0.2 |
| v45 | 0.0 | 0.0 | 0.1 | 0.0 | 0.0 | 0.0 | 0.0 | 0.0 | 0.1 | 0.1 | 0.0 | 0.0 | 0.0 | 0.0 | 0.2 | 0.2 |
| v46 | 0.0 | 0.0 | 0.1 | 0.1 | 0.0 | 0.0 | 6.0 | 5.3 | 0.0 | 0.0 | 0.0 | 0.0 | 0.2 | 0.2 | 0.0 | 0.0 |
| v47 | 144.1 | 0.7 | 139.1 | 0.5 | 139.8 | 0.1 | 120.6 | 0.1 | 149.7 | 0.6 | 127.0 | 0.6 | 101.3 | 0.8 | 128.0 | 0.4 |
| v48 | 0.1 | 0.1 | 1.1 | 1.0 | -3.0 | 0.1 | -10.2 | 0.0 | 0.0 | 0.0 | 0.0 | 0.0 | -9.9 | 0.1 | -7.5 | 0.2 |
| v49 | 10.4 | 0.0 | 13.8 | 0.0 | 4.0 | 0.0 | 5.2 | 0.0 | 11.0 | 0.0 | 10.7 | 0.0 | 47.7 | 2.3 | 27.6 | 0.3 |
| v50 | 178.3 | 5.3 | 184.9 | 2.2 | 211.1 | 1.3 | 171.7 | 1.4 | 206.3 | 1.9 | 204.3 | 0.5 | 167.6 | 0.6 | 192.8 | 0.4 |
